# Anticardiolipin and/or anti-β2-glycoprotein-I antibodies are associated with adverse IVF outcomes

**DOI:** 10.3389/fimmu.2022.986893

**Published:** 2022-11-02

**Authors:** Li Wu, Xuhui Fang, Fangting Lu, Yu Zhang, Yanshi Wang, Joanne Kwak-Kim

**Affiliations:** ^1^ Reproductive Medicine Center, Department of Obstetrics and Gynecology, The First Affiliated Hospital of University of Science and Technology of China (USTC), Division of Life Sciences and Medicine, University of Science and Technology of China, Hefei, Anhui, China; ^2^ Reproductive Medicine and Immunology, Obstetrics and Gynecology, Clinical Sciences Department, Chicago Medical School, Rosalind Franklin University of Medicine and Science, Vernon Hills, IL, United States; ^3^ Center for Cancer Cell Biology, Immunology and Infection Diseases, Chicago Medical School, Rosalind Franklin University of Medicine and Science, North Chicago, IL, United States

**Keywords:** anticardiolipin, anti-β2-glycoprotein-I, antiphospholipid syndrome, IVF outcomes, pregnancy outcome

## Abstract

**Objective:**

The purpose of the study is to evaluate the effects of anticardiolipin (aCL) and/or anti-β2-glycoprotein-I (aβ2GPI) antibodies, namely antiphospholipid antibodies (aPL), on *in vitro* fertilization (IVF) outcomes.

**Materials and methods:**

The study group comprised infertile women with aPL undergoing IVF-ET cycles. Controls were infertile women with tubal etiology without aPL. The impact of aPL on reproductive outcomes, such as oocyte quality, embryo quality, and implantation capacity, was compared between the study group and controls. Additionally, peripheral blood T cell subsets, such as T helper (Th)1, Th2, Th17, and T regulatory (Treg) cells and cytokines, were analyzed by the flow cytometry. Differences between the study group and controls were analyzed.

**Results:**

A total of 132 infertile women, including 44 women with aPL, and 88 controls were sequentially recruited for this study. Women with aPL had lower numbers of total and perfect/available embryos and lower rates of MII oocytes, blastocyst formation, perfect and available embryos, implantation, clinical pregnancy, and take-home baby. Additionally, imbalanced Th1/Th2 and Th17/Treg ratios, significantly higher levels of serum IL-2, TNF-α, IFN-γ, and IL-17A, and a significantly lower serum IL-4 were noticed in women with aPL compared to controls.

**Conclusion:**

Women with aPL such as aCL and/or aβ2GPI antibodies were associated with adverse IVF outcomes. Early screening for aPL and appropriate consultation for couples undergoing IVF should be considered. In addition, underlying immunopathology and inflammatory immune mechanisms associated with aPL should be further explored.

## Introduction

Anticardiolipin (aCL) and anti-β2-glycoprotein-I (aβ2GPI) antibodies, as well as lupus anticoagulant (LA), belong to antiphospholipid antibodies (aPL), a family of heterogeneous autoantibodies directed against phospholipids and phospholipid-binding proteins (1). The presence of persistent serum aPL positivity, venous/arterial thrombosis and obstetric complications are the main characteristics of antiphospholipid syndrome (APS) (2). As one of the autoimmune diseases, APS is closely associated with adverse pregnancy outcomes, such as recurrent pregnancy losses (RPL), preeclampsia, intrauterine growth restriction, and preterm delivery (1, 3, 4). The incidence and prevalence of APS are relatively low, estimated to be about ~5 new cases per 100,000 individuals per year and ~40-50 cases per 100,000 individuals, respectively. However, the seroprevalence of aPL in the general population was as high as 1%~5% (3, 5).

Infertile women often present aPL positivity. The prevalence of positive aPL has been reported to be higher in infertile women (15-53%) than in normal fertile women (1-3%); 3.3% to 23.7% in unexplained infertility, and 0% to 66% in women undergoing IVF (6–8). However, most of them do not meet the diagnostic criteria of APS. APL, such as aCL and aβ2GPI antibodies, have been reported to play a central role in the pathogenesis of APS (8, 9). The presence of aPL is a precondition. Indeed, thrombosis associated with APS results from the second hit by innate inflammatory immune responses, often leading to recurrent obstetrical complications. β2GPI-dependent aPL are thought to recognize their antigen on placental tissues, inhibit the growth and differentiation of trophoblasts, and eventually cause defective placentation (10).

Whether the aPL positivity in women without APS affects the subsequent IVF outcomes has not been studied well. Therefore, the present study aimed to investigate the impact of aPL (aCL and aβ2GPI antibodies) on IVF outcomes. Markers of oocyte quality (number of oocytes, MII oocyte rate, and normal fertilization rate), embryo quality (number of embryos, perfect and available embryo rates, and blastocyst formation rate), and implantation capacity (implantation rate, clinical pregnancy rate, miscarriage rate, and take home baby rate) were investigated. In addition, the immune-inflammatory status of aPL-positive women, including peripheral blood Th cell subsets and serum cytokine levels, were investigated. This study affirms whether aPL should be investigated in women undergoing infertility treatment.

## Materials and methods

### Study population

**Figure 1:**
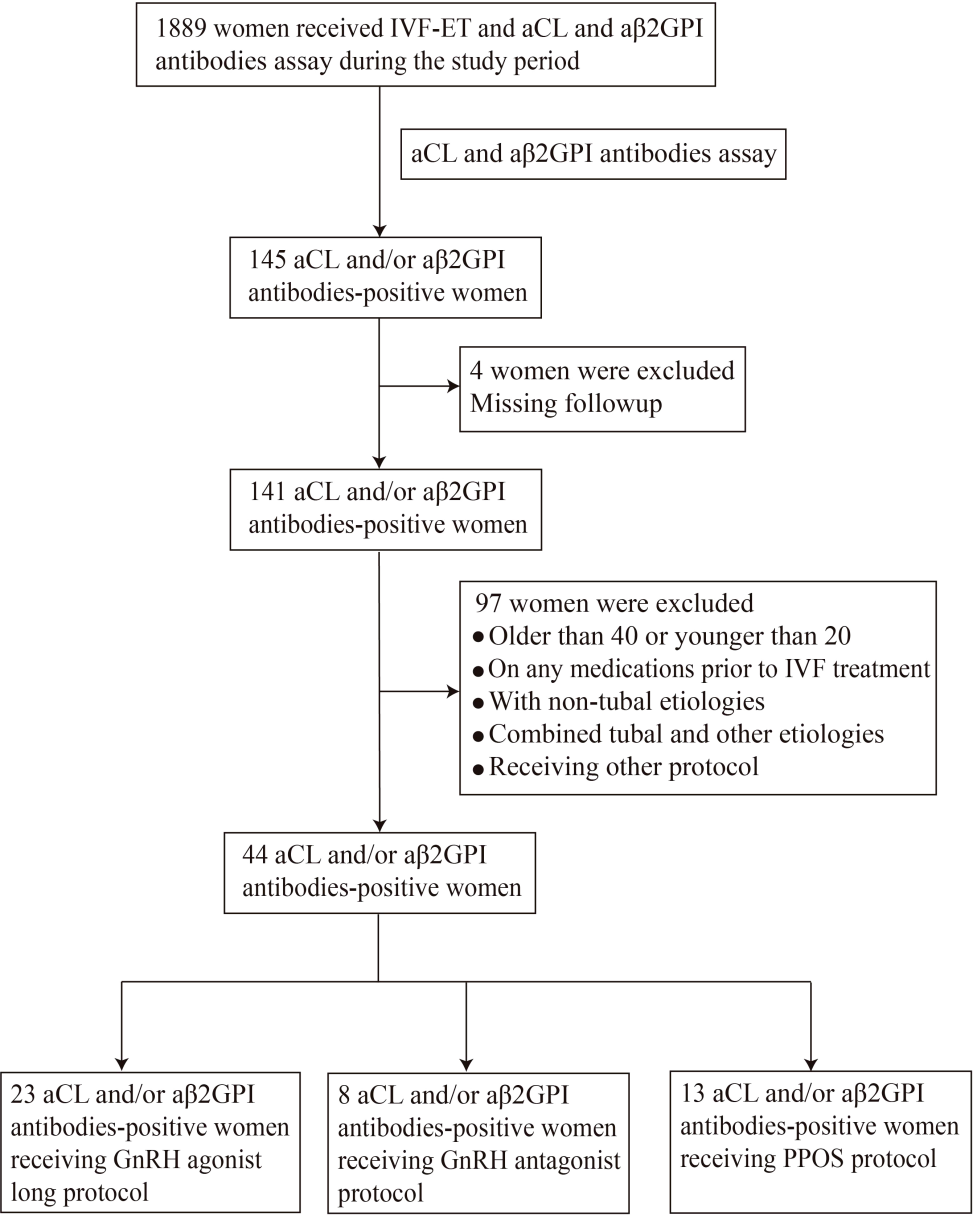
The patient selection scheme for aCL and/or aβ2GPI antibody (aPL) positive women.

Infertile women undergoing IVF-ET cycles were recruited at the Reproductive Medicine Center, Department of Obstetrics and Gynecology, the First Affiliated Hospital of USTC from July 2019 to May 2021. This study was approved by the Ethics Committee of Anhui Provincial Hospital (Approval No. 2021-RE-112). All study participants signed a consent form prior to entering the study. A total of 1889 infertile women who underwent IVF-ET cycles during the study period were screened for aPL. Women who met the selection criteria were sequentially enrolled in the study, including 44 aCL and/or aβ2GPI antibodies-positive and 88 antibodies-negative control women (**Figure 1**).

The inclusion criteria of the study group were women who were older than 20 years and younger than 40 years, 2) with positive aPL (aCL and/or aβ2GPI antibody), 3) with infertility of tubal etiology, and 4) without any medications before the first IVF cycle. The exclusion criteria for both study and control groups were followings; 1) women underwent intracytoplasmic sperm injection (ICSI), 2) infertility with chromosomal or anatomical abnormalities, 3) presence of autoimmune diseases (such as APS, systemic lupus erythematosus, Sjögren’s syndrome and autoimmune thyroid disease, etc.) or 4) presence of endometriosis or polycystic ovary syndrome. The control group was composed of infertile women undergoing IVF-ET cycles during the same period with tubal etiology and without aPL. Controls were both age and body mass index (BMI) matched with the study group.

In order to exclude the interference of different ovulation induction protocols on the results, the study group and the control group were further divided into the GnRH agonist protocol group (23 women with aPL and 48 controls), GnRH antagonist protocol group (8 women with aPL and 28 controls) and PPOS protocol group (13 women with aPL and 12 controls) as reported prior (11).

### aCL and aβ2GPI antibodies assay

Serum aCL and aβ2GPI antibodies were measured by enzyme-linked immunosorbent assays (ELISAs) (EUROIMMUN, Lübeck, Germany). In brief, the peripheral blood sample was examined in three wells, two wells with antigen and one without, to subtract the background from the specific binding. According to the instructions of the ELISA kit, cutoff levels were calculated at the 99th percentile. IgG, IgM, and IgA subtypes of aCL and aβ2GPI >20 CU confirmed at the 12-week interval were considered positive.

### Th1, Th2, Th17, and Treg cells assay

On the third day of the menstrual period, 5 mL of venous blood per woman was drawn from the antecubital vein with the vacuum anticoagulant blood collection tubes. T cell subsets and serum cytokine levels were analyzed by Flow Cytometry within 6 hours after venipuncture. In brief, peripheral blood mononuclear cells (PBMCs) were isolated using density-gradient centrifugation (250g, 6min) and incubated with 5% CO_2_ at 37˚C for 4~5 h. Following surface staining, cells were fixed and permeabilized with a Fixation/Permeabilization Solution kit (BD Bioscience), where intracellular staining was performed. The corresponding fluorescent antibodies labeled T cell subsets: anti-human CD3 phycoerythrin (PE)-cy7, anti-human CD4 peridinin chlorophyll protein (PerCP-cy5.5), anti-human IFN-γ fluorescein isothiocyanate (FITC), anti-human IL-4 BV421, anti-human IL-17A PE, anti-human CD25 BV421 and anti-human Foxp3 allophycocyanin (APC). All antibodies were purchased from BD Biosciences. Th1 cells (CD3^+^CD4^+^IFN-γ^+^), Th2 cells (CD3^+^CD4^+^IL-4^+^), Th17 cells (CD3^+^CD4^+^IL-17A^+^) and Treg cells (CD3^+^CD4^+^CD25^high^Foxp3^+^) were detected with Beckman flow cytometer (Beckman Coulter, CA, USA) and analyzed by FlowJo software (FlowJo, LLC). Isotype controls were used as negative controls.

### Cytokines assay

The serum cytokines, including IL-2, IL-4, IL-6, IL-10, TNF-α, IFN-γ, and IL-17A, were measured by flow cytometry with a Cytometric Bead Array (CBA) kit (BD Biosciences). The peripheral blood samples were collected on cycle day 3 and incubated with microspheres of different fluorescence intensities conjugated with corresponding captured antibodies and PE fluorescent-labeled antibodies specific for each cytokine simultaneously. After vortexing the sample, the captured microsphere mixture was centrifuged at 200g for 5 min, and the supernatant was discarded. Then, an equal amount of microsphere buffer was added to resuspend microspheres and incubated in the dark for 15 min. Next, equal amounts of sample, capture microspheres, and fluorescence detection reagent were mixed and incubated at room temperature for 2.5 h. After incubation, the supernatant was discarded after centrifugation at 200g for 5 min and resuspended in 200μl phosphate buffer saline (PBS). Cytokine concentrations were measured with the Beckman flow cytometer (Beckman Coulter, CA, USA) and analyzed by FlowJo software (FlowJo, LLC). Standard curves were developed according to the manufacturer’s instructions.

### GnRH agonist long protocol

3.75mg long-acting GnRH agonist triptorelin (Ipsen Pharma Biotech, France) was injected intramuscularly cycle day 2 or 5 after ensuring no dominant (>10mm) follicles or ovarian cysts. After one month, the pituitary gland was completely downregulated. Then, 150~225 IU of recombinant human FSH (rFSH) (Gonal-F^®^, Merck Serono, Switzerland) was initiated for the first 5 days, and then the rFSH dose was adjusted individually according to the ovarian response. In the late-follicle phase, recombinant LH 75 IU (Luveris^®^, Merck Serono, Switzerland, 75IU injection daily) was added.

### GnRH antagonist protocol

rFSH 150~300 IU was injected on cycle day 2 or 3 after ensuring no dominant (>10mm) follicles or ovarian cysts. Subsequently, according to the ovarian response assessed by transvaginal ultrasound and serum hormone levels, the rFSH dose was adjusted, and HMG (75U/day) or r-LH was added. GnRH antagonist 0.25mg (Cetrotide^®^, Merck Serono, Switzerland) was applied when follicles reached 13~14 mm or serum E2≥500 pg/mL until the day of hCG injection.

### PPOS protocol

On cycle day 3, medroxyprogesterone acetate (MPA, Xianju, Zhejiang, 4 mg/tablet, 1~3 tablets/day) was used until the day of ovulation induction. FSH or HMG 100~300 U was injected daily simultaneously, and the dose was adjusted according to the results of vaginal ultrasound and reproductive hormones.

### Egg retrieval, embryo quality assessment, and embryo transfer

For all protocols, when the diameter of two or three follicles reached 17mm, or at least one follicle reached 18mm, 250μg recombinant human chorionic gonadotropin (hCG, Ovitrelle^®^, Merck Serono, Switzerland) was injected. Oocyte retrieval was performed 36 hours after hCG administration. The quality of the embryos was evaluated by the number, size, and degree of blastomere fragmentation on the third day after oocyte retrieval, as described previously (12). Embryo quality is primarily assessed by our center’s day 3 embryo grading system. Day-3 embryos were evaluated based on the number and size of their blastomeres and the degree of fragmentation; Grade 1, 6–8 even, equally sized blastomeres without fragmentation of the blastomeres; Grade 2, 6–8 even, equally sized blastomeres, and less than 20% fragmentation of the blastomeres; Grade 3, 4–6 uneven or irregularly shaped blastomeres, and 20-50% fragmentation of the blastomeres; Grade 4, the embryos are considered non-viable with more than 50% fragmentation or with even lysed, contracted, or dark blastomeres. Embryos with grade 1 and 2 were considered good-quality or perfect embryos, and embryos with grade 1, 2, and 3 were considered available embryos. One or two perfect embryos were transferred during the ET cycle.

### The aPL treatments

Patients with aPL received at least two consecutive IVF-ET cycles. During the first cycle, no treatment for aPL was provided. Patients who failed the first IVF cycle or succeeded with low-quality embryos were tested for aPL antibodies before the second ET cycle. Women with positive aPL administrated low-dose aspirin (LDA, 100 mg, orally, daily) and hydroxychloroquine sulfate (100mg, twice daily) treatment for 1-3 months before the second IVF cycle. The same ovulation induction protocol was used for the second cycle. After the embryo transfer, low molecular weight heparin (LMWH, 4000 U, daily, subcutaneously) was initiated for women with aPL.

### Statistical analysis

For T cell subsets and cytokine assays, two replicates were tested for one sample. The sample sizes were calculated using PASS 15 (NCSS Statistical Software, LLC, Kaysville, Utah, USA). Differences were compared using the student t-test for normally distributed continuous variables, the χ2 test for categorical variables, and the Mann-Whitney U test for non-parametric variables. All statistical analyses were performed C SPSS 22.0 (SPSS, Inc., Chicago, IL, USA). Statistical significance was defined when a *p*-value was less than 0.05 (two-tailed).

## Results

### Composition and distribution of aCL and/or aβ2GPI antibodies

**Figure 2:**
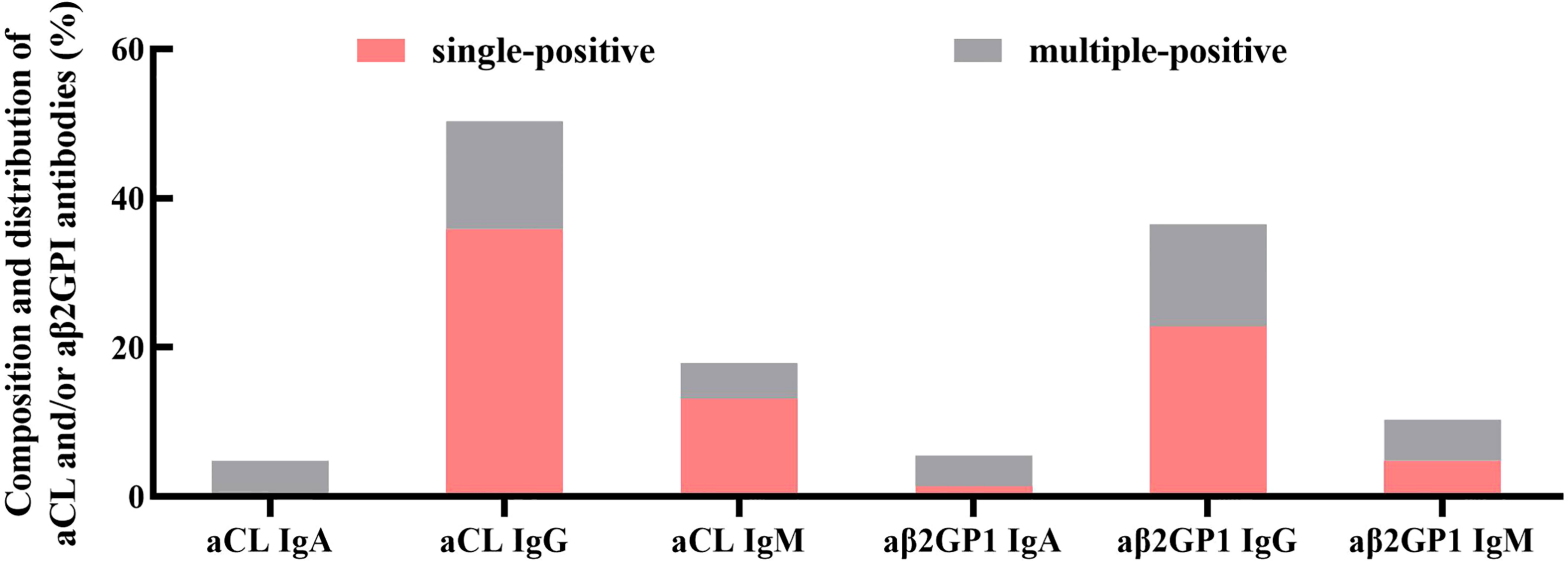
Composition and distribution of aCL and/or aβ2GPI antibodies (aPL). The most frequent antibody class was IgG, followed by IgM. The least prevalent antibody class was IgA. Additionally, multiple antibody-positive women accounted for 22.07% of all aPL-positive women.

A total of 1,889 patients received aPL assay (IgA, IgG, and IgM isotypes) and IVF-ET cycles during the study period. The prevalence of positive aCL and/or aβ2GPI antibodies was 7.68% (n=145). The composition and distribution of aCL and/or aβ2GPI antibodies were summarized in **Figure 2**. The most frequent antibody subtype was IgG aCL, including IgG aCL positive only (n=52, 35.86%) and IgG aCL with IgM and/or IgA positive (n=73, 50.34%), and the second most frequent antibody subtype was IgG aβ2GPI; IgG aβ2GPI (n=53) was 36.55% and IgG aβ2GPI only (n=33) was 22.76%. This was followed by IgM aCL (IgM aCL, n=26, 17.93%; IgM aCL only, n=19, 13.10%) and IgM aβ2GPI (IgM aβ2GPI, n=15, 10.34%; IgM aβ2GPI only, n=7, 4.83%). The least prevalent antibody was IgA aCL (IgA aCL, n=7, 4.83%; IgA aCL only, n=0, 0%) and IgA aβ2GPI (IgA aβ2GPI, n=8, 5.52%; IgA aβ2GPI only, n=2, 1.38%). Additionally, the frequency of women with multiple antibodies such as both ACA and aβ2GPI was 22.07% (n=32).

### Baseline and clinical characteristics

As shown in **Table 1**, there were no statistical differences between antibody-positive women and the control group in age, BMI, duration of infertility, basal hormonal levels (anti-Müllerian hormone (AMH), FSH, LH, E2, prolactin (PRL)), hormone levels on the day of hCG trigger, thyroid-related hormones (thyroid-stimulating hormone (TSH), free T4 (FT4), and free T3 (FT3)), fasting blood glucose (FBG), fasting insulin (INS), cancer antigen 125 (CA125), D-Dimer (D-D), homocysteine (HCY), and serum 25(OH)VD levels.

**Table 1:** Baseline of clinical and laboratory characteristics of women with tubal etiology infertility with and without antiphospholipid antibodies (aPL, aCL and/or aB2GPi antibodies).

Characteristics	aPL positive (n=44)	aPL negative (n=88)	*p*-value
Age (years)	30.9 ± 2.7	30.4 ± 3.5	0.466
Duration of infertility (years)	3.3 ± 2.2	2.9 ± 2.0	0.324
BMI (kg/m^2^)	23.0 ± 2.9	22.5 ± 2.8	0.314
AMH (ng/ml)	3.9 ± 3.1	4.3 ± 2.7	0.644
Basal FSH (IU/L)	6.2 ± 1.7	6.6 ± 1.4	0.279
Basal LH (IU/L)	4.3 ± 1.8	4.0 ± 1.6	0.408
Basal E2 (pg/ml)	46.6 ± 17.3	43.2 ± 17.6	0.395
PRL (ng/ml)	13.9 ± 6.0	12.6 ± 4.8	0.262
LH level on hCG day (IU/L)	1.4 ± 0.9	1.6 ± 0.7	0.228
E2 level on hCG day (pg/ml)	2901.0 ± 1472.3	3189.3 ± 1335.8	0.259
P level on hCG day (ng/ml)	0.9 ± 0.6	0.9 ± 0.4	0.753
TSH (mIU/L)	2.1 ± 0.8	2.2 ± 0.8	0.734
FT4 (pmol/L)	17.1 ± 2.8	17.2 ± 2.7	0.810
FT3 (pmol/L)	4.9 ± 0.7	5.0 ± 0.6	0.578
FBG (mmol/L)	5.1 ± 0.7	5.0 ± 0.3	0.336
INS (pmol/L)	71.1 ± 31.9	67.1 ± 28.8	0.350
CA125 (U/ml)	15.5 ± 5.3	15.3 ± 5.5	0.856
D-D(ug/ml)	0.4 ± 0.2	0.4 ± 0.2	0.372
HCY (umol/L)	6.9 ± 1.5	6.5 ± 1.8	0.276
Vitamin D level (ng/ml)	15.7 ± 4.6	17.1 ± 5.5	0.165

The data were represented by mean ± SD unless otherwise stated. P-value <0.05 was considered to be statistically significant.

BMI, body mass index; AMH, anti-Müllerian hormone; FSH, follicle-stimulating hormone; LH, luteinizing hormone; E2, estradiol; PRL, prolactin; TSH, thyroid stimulating hormone; FT4, free tetraiodothyronine; FT3, free triiodothyronine; FBG, fasting blood glucose; INS, fasting insulin; CA125, cancer antigen 125; D-D, D-Dimer; HCY, homocysteine.

### Peripheral blood Th1, Th2, Th17, and Treg cells

**Figure 3:**
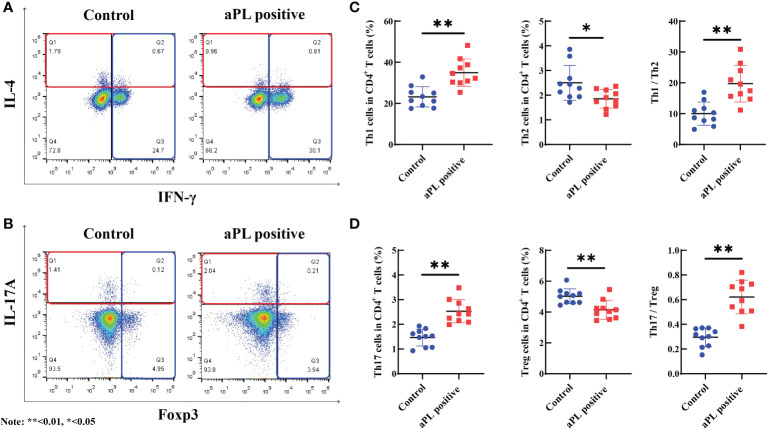
The comparison of peripheral blood Th1, Th2, Th17, and Treg cells, and Th1/Th2 and Th17/Treg ratios in women with positive (n=10) and negative (n=10) aCL and/or aβ2GPI antibodies (aPL). Women with aPL had significantly higher levels of peripheral blood Th1 and Th17 cells and significantly lower levels of peripheral blood Th2 and Treg cells than controls. Th1/Th2 and Th17/Treg ratios were significantly higher in women with aPL than in controls. **(A)** The proportion of Th1 and Th2 cells in women with aPL and controls; **(B)** The proportion of Th17 and Treg cells in women with aPL and controls; **(C)** Comparison of the proportion of Th1, Th2, and Th1/Th2 ratio between women with aPL and control; **(D)** Comparison of the proportion of Th17, Treg, and Th17/Treg ratio between women with aPL and controls. **P<0.01, *P<0.05.

When compared with the control group (n=10), aPL-positive women (n=10) had significantly higher percentages of peripheral blood Th1 (34.9 ± 6.7% versus 23.2 ± 4.9%, *p*=0.000) and Th17 cells (2.5 ± 0.5% versus 1.5 ± 0.3%, *p*=0.000) and significantly lower proportions of peripheral blood Th2 (1.9 ± 0.4% versus 2.5 ± 0.7%, *p*=0.019) and Treg cells (4.1 ± 0.6% versus 5.0 ± 0.5%, *p*=0.002) (**Figure 3**). Additionally, Th1/Th2 (19.7 ± 6.0 versus 10.0 ± 3.7, *p*=0.000) and Th17/Treg (0.6 ± 0.1 versus 0.3 ± 0.1, *p*=0.000) ratios were significantly higher in women with aPL antibodies than controls.

### Serum cytokines levels

**Figure 4:**
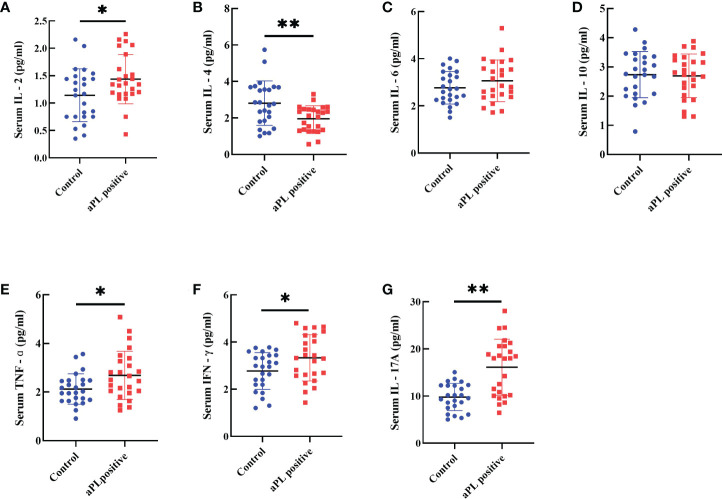
Comparison of serum levels including IL-2 **(A)**, IL-4 **(B)**, IL-6 **(C)**, IL-10 **(D)**, TNF-α **(E)**, IFN-g **(F)**, and IL-17A **(G)** in women with positive (n=25) and negative (n=25) aCL and/or aβ2GPI antibodies (aPL). Women with aPL had significantly higher levels of serum IL-2, TNF-α, IFN-γ, and IL-17A and significantly lower serum IL-4 than controls. However, IL-6 and IL-10 levels were not different. **P<0.01, *P<0.05.

The aPL-positive women (n=25) had significantly higher levels of serum IL-2 (1.4 ± 0.4 versus 1.1 ± 0.5 pg/ml, *p*=0.031), TNF-α (2.7 ± 1.0 versus 2.1 ± 0.6 pg/ml, *p*=0.021), IFN-γ (3.3 ± 1.0 versus 2.8 ± 0.8 pg/ml, *p*=0.030) and IL-17A (16.1 ± 6.0 versus 9.8 ± 2.9 pg/ml, *p*=0.000), and significantly lower levels of serum IL-4 (2.0 ± 0.7 versus 2.8 ± 1.2 pg/ml, *p*=0.004) than those of the control group (n=25) (**Figure 4**). However, there were no significant differences in concentrations of serum IL-6 and IL-10.

### IVF laboratory outcomes between antibodies-positive women and the control group

The details of IVF outcomes between women with positive aPL and the control group are present in **Table 2**. There were no significant differences in days of ovarian stimulation, total Gn dose, and retrieved oocytes among different ovulation induction protocols. However, aPL-positive women had significantly lower numbers of embryos and perfect/available embryos and lower M II oocyte rate, normal fertilization rate, blastocyst formation rate, and perfect/available embryo rate than controls. However, M II oocytes and perfect/available embryo rates did not reach statistical significance in women with the antagonist protocol.

**Table 2:** Reproductive outcomes of IVF cycles in women with tubal etiology infertility with and without APA.

IVF laboratory outcomes	Agonist protocol			Antagonist protocol			PPOS protocol		
	aPL positive (n=23)	aPL negative (n=48)	p-value	aPL positive (n=8)	aPL negative (n=28)	p-value	aPL positive (n=13)	aPL negative (n=12)	p-value
Days of ovarian stimulation	11.8 ± 2.0	11.6 ± 2.2	0.743	9.0 ± 2.6	10.5 ± 1.7	0.106	10.1 ± 3.1	9.6 ± 1.7	0.627
Total Gn dose (75IU/each)	2310.0 ± 1103.5	2171.9 ± 1021.1	0.609	1875.0 ± 826.7	1935.7 ± 765.4	0.876	2127.0 ± 889.1	1868.8 ± 786.9	0.468
Retrieved oocytes	12.1 ± 6.3	13.5 ± 4.5	0.257	8.6 ± 3.7	11.4 ± 5.5	0.210	5.0 ± 3.5	6.5 ± 3.2	0.290
MII oocytes rate (%)	67.1 ± 24.0	81.6 ± 11.4	**0.004**	64.5 ± 26.1	83.0± 13.3	0.114	74.3 ± 18.0	92.7 ± 12.1	**0.009**
Normal fertilization rate (%)	56.0 ± 27.1	71.9 ± 12.0	**0.006**	54.1 ± 25.5	73.4 ± 18.2	**0.027**	63.8 ± 25.1	85.1 ± 17.3	**0.024**
Embryos	7.8 ± 4.1	10.8 ± 3.4	**0.001**	5.4 ± 3.5	8.8 ± 3.9	**0.044**	3.1 ± 1.9	6.0 ± 2.9	**0.011**
Perfect embryos	2.5 ± 1.8	6.3 ± 2.1	**0.000**	1.9 ± 1.2	6.3 ± 3.6	**0.003**	1.7 ± 2.6	5.3 ± 2.4	**0.002**
Available embryos	4.1 ± 3.0	8.2 ± 2.2	**0.000**	2.6 ± 1.7	7.1 ± 4.0	**0.007**	2.6 ± 4.5	5.8 ± 2.9	**0.048**
Blastocyst formation rate (%)	22.5 ± 22.7	41.1 ± 14.0	**0.000**	20.2 ± 14.5	46.2 ± 19.6	**0.017**	16.3 ± 17.4	42.5 ± 16.5	**0.001**
Perfect embryo rate (%)	38.1 ± 23.2	71.0 ± 18.3	**0.000**	47.3 ± 38.3	73.9 ± 21.6	0.121	21.7 ± 24.2	89.9 ± 12.9	**0.000**
Available embryo rate (%)	53.2 ± 31.3	88.8 ± 11.5	**0.000**	60.0 ± 37.1	82.6 ± 20.3	0.165	36.3 ± 37.8	86.1 ± 19.0	**0.001**

aPL, antiphospholipid antibody, such as anti-cardiolipin antibody and/or β2-glycoprotein-I antibody; PPOS, progestin-primed ovarian stimulation protocol; MII oocytes rate, the number of MII oocytes / the number of total oocytes; Blastocyst formation rate, the number of blastocysts / the number of normally fertilized embryos; Normal fertilization rate, the number of 2PNs / the number of cumulus-oocyte complexes; Perfect embryo rate, (the number of Grade 1 and Grade 2 embryos) / the number of 2PNs; Available embryo rate, (the number of Grade 1, Grade 2 and Grade 3 embryos) / the number of 2PNs. P-value <0.05 was considered to be statistically significant.

### IVF laboratory outcomes between antibodies-positive women and the treatment group

The IVF outcomes of aPL-positive women without and with hydroxychloroquine, LDA and LMWH treatment are shown in **Table 3**. There were no significant differences in days of ovarian stimulation, total Gn dose, and the numbers of retrieved oocytes and embryos. The number of perfect/available embryos, perfect/available embryo rate, M II oocyte rate, blastocyst formation rate, and normal fertilization rate were all higher in the treatment group than in the controls. In agonist protocol, the number of perfect/available embryos, perfect/available embryo rate, blastocyst formation rate, and normal fertilization rate were significantly higher in the treatment group than in the non-treated group. In antagonist protocol, the number of perfect/available embryos and perfect/available embryo rate were significantly increased. In PPOS protocol, the perfect/available embryo rate was significantly increased compared to controls.

**Table 3:** Reproductive outcomes of IVF cycles in aPL positive women before and after hydroxychloroquine and antiplatelets treatment.

IVF laboratory outcomes	Agonist protocol			Antagonist protocol			PPOS protocol		
	Before treatment (n=23)	After treatment (n=21)	*p*-value	Before treatment (n=8)	After treatment (n=8)	*p*-value	Before treatment (n=13)	After treatment (n=12)	*p*-value
Days of ovarian stimulation	11.8 ± 2.0	11.4 ± 2.3	0.649	9.0 ± 2.6	10.0 ± 1.2	0.454	10.1 ± 3.1	9.8 ± 2.6	0.776
Total Gn dose (IU)	2310.0 ± 1103.5	2525.0 ± 1297.0	0.681	1875.0 ± 826.7	1743.8 ± 824.7	0.818	2127.0 ± 889.1	2131.3 ± 646.3	0.804
Retrieved oocytes	12.1 ± 6.3	13.1 ± 6.1	0.662	8.6 ± 3.7	11.3 ± 7.9	0.433	5.0 ± 3.5	6.7 ± 4.6	0.341
M II oocytes rate (%)	67.1 ± 24.0	74.8 ± 17.6	0.236	64.5 ± 26.1	83.2 ± 34.1	0.271	74.3 ± 18.0	85.1 ± 14.8	0.112
Normal fertilization rate (%)	56.0 ± 27.1	72.1 ± 15.1	**0.026**	54.1 ± 25.5	64.1 ± 19.5	0.462	63.8 ± 25.1	75.1 ± 20.5	0.229
Embryos	7.8 ± 4.1	8.8 ± 3.0	0.525	5.4 ± 3.5	7.6 ± 4.4	0.335	3.1 ± 1.9	5.5 ± 3.7	0.062
Perfect embryos	2.5 ± 1.8	5.4 ± 1.8	**0.000**	1.9 ± 1.2	5.1 ± 3.1	**0.022**	1.7 ± 2.6	3.2 ± 2.6	0.143
Available embryos	4.1 ± 3.0	6.8 ± 3.1	**0.034**	2.6 ± 1.7	6.3 ± 3.0	**0.015**	2.6 ± 4.5	4.4 ± 4.0	0.300
Blastocyst formation rate (%)	22.5 ± 22.7	41.0 ± 14.7	**0.004**	20.2 ± 14.5	36.4 ± 14.7	0.125	16.3 ± 17.4	22.5 ± 20.5	0.426
Perfect embryo rate (%)	38.1 ± 23.2	65.6 ± 13.8	**0.003**	47.3 ± 38.3	77.3 ± 23.3	0.102	21.7 ± 24.2	61.8 ± 31.1	**0.002**
Available embryo rate (%)	53.2 ± 31.3	80.0 ± 16.0	**0.000**	60.0 ± 37.1	98.8. ± 18.1	**0.035**	36.3 ± 37.8	77.8 ± 29.2	**0.005**

aPL, antiphospholipid antibody, such as anti-cardiolipin antibody and/or β2-glycoprotein-I antibody. P-value <0.05 was considered to be statistically significant.

### IVF outcomes

**Figure 5:**
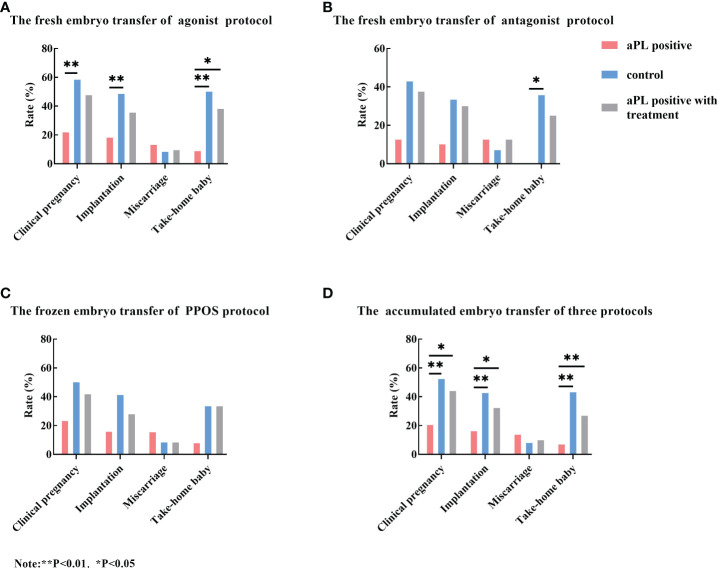
Comparison of the clinical pregnancy rate, implantation rate, miscarriage rate, and take-home baby rate among aCL and/or aβ2GP1 antibody (aPL) positive women without treatment (the aPL group), aPL negative women (the control group), and aPL-positive women with treatment (the aPL treatment group). Regardless of stimulation protocol, the clinical pregnancy rate, implantation rate, and take-home baby rates of the control and aPL treatment groups were higher than those of the aPL group. When comparing the total embryo transfer outcomes of three protocols, the clinical pregnancy rate, implantation rate, and take-home baby rate of the control and the treatment groups were significantly higher than those of the aPL group. However, the miscarriage rates were not different among the three groups. **(A)** The fresh embryo transfer outcomes of GnRH agonist protocol; **(B)** The fresh embryo transfer outcomes of GnRH antagonist protocol; **(C)** The frozen embryo transfer outcomes of PPOS protocol; and **(D)** The total embryo transfer outcomes of three protocols. **P<0.01, *P<0.05.

Data concerning IVF outcomes of aPL-positive women without treatment, the control group, and the treatment group were summarized in **Figure 5**. Regardless of the IVF protocol (agonist protocol, antagonist protocol, or PPOS protocol), the clinical pregnancy rate, implantation rate, and take home baby rate were lower in aPL-positive women compared with the control and the treatment groups. However, the IVF outcomes of the control group were not significantly different from those of antagonist and PPOS protocols (**Figures 5A–C**).

When the accumulated embryo transfer outcomes were compared, the clinical pregnancy rate, implantation rate, and take-home baby rate of the control and the treatment groups were significantly higher than those of the aPL-positive group. However, the miscarriage rate did not reach statistical significance (**Figure 5D**). However, the IVF outcomes of each protocol were not different with the treatment as compared with those without treatment.

### IVF laboratory outcomes and pregnancy outcomes between women with single antibody-positive and multiple antibody-positive

When compared with the single aPL-positive women (One subtype positive of aCL or aβ2GPI positivity), multiple aPL-positive (At least two subtype positive of aCL or aβ2GPI positivity) women had lower M II oocytes rate, normal fertilization rate, blastocyst formation rate, perfect and available embryo rates, implantation rate, clinical pregnancy rate, and take-home baby rate. Yet, all of them did not reach a statistically significant level, possibly due to the small size of the study population (**Table 4**).

**Table 4:** Reproductive outcomes of IVF cycles in women with single APA vs. both APA antibodies in women with tubal etiology infertility.

IVF laboratory outcomes and pregnancy outcomes	Single antibody-positive (n=36)	Both antibody-positive (n=8)	*p*-value
Days of ovarian stimulation	11.0 ± 3.7	11.1 ± 2.4	0.928
Total Gn dose (IU)	2151.2 ± 1045.0	2437.5 ± 868.4	0.480
Retrieved oocytes	10.4 ± 6.3	9.7 ± 3.0	0.676
M II oocytes rate (%)	71.9 ± 19.1	53.9 ± 32.0	0.139
Normal fertilization rate (%)	59.6 ± 25.3	43.3 ± 32.2	0.105
Embryos	6.6 ± 3.7	5.7 ± 3.9	0.583
Perfect embryos	2.4 ± 2.3	1.5 ± 1.4	0.336
Available embryos	3.4 ± 3.3	3.0 ± 2.7	0.802
Blastocyst formation rate (%)	21.4 ± 20.6	16.0 ± 20.5	0.537
Perfect embryo rate (%)	33.9 ± 24.4	23.9 ± 22.5	0.354
Available embryo rate (%) Clinical pregnancy rate (%) Implantation rate (%) Miscarriage rate (%) Take-home baby rate (%)	48.8 ± 33.1 20.4 (11/54) 13.3 (11/83) 25.9 (14/54) 11.1 (6/54)	41.2 ± 41.7 13.3 (2/15) 8.7 (2/23) 40 (6/15) 6.7 (1/15)	0.592 0.808 0.818 0.459 0.983

APA, antiphospholipid antibody, such as anti-cardiolipin antibody and/or β2-glycoprotein-I antibody; Clinical pregnancy rate, the number of clinical pregnancy cycles / the total cycles; Implantation rate, the number of gestational sacs / the number of embryos transferred; Miscarriage rate, the number of miscarriage cycles / the total cycles; Take-home baby rate, the number of take-home baby cycles / the total cycles.

## Discussion

The aPL can be detected in patients with APS, systemic lupus erythematosus, rheumatoid arthritis, infections, and even healthy individuals (13, 14). Although the effects of aPL on obstetrical complications and pregnancy outcomes have been reported (1, 3, 4), the impacts of aPL on IVF outcomes have not been systematically investigated. In our study, we report that aCL and aβ2GPI antibodies are associated with adverse IVF outcomes, including significantly lower numbers of perfect and available embryos, lower normal fertilization rate, blastocyst formation rate, perfect and available embryo rates, clinical pregnancy rate, implantation rate, and take-home baby rate.

The aPL are the key hallmark of APS and can activate numerous cells, including endothelial cells, monocytes, and neutrophils, leading to alteration in immunity and inflammation (9). Therefore, we further investigated the immunological and inflammatory status by measuring the proportions of circulating Th1, Th2, Th17, and Treg cells and serum cytokine levels in women with aPL (positive aCL and/or aβ2GPI antibodies). Our study demonstrated that in women with positive aPL, the proportions of Th1 and Th17 cells in peripheral blood were significantly increased, while the Th2 and Treg cell proportions were significantly decreased. Additionally, the ratios of Th1/Th2 and Th17/Treg were unbalanced in aPL-positive women, which was consistent with previous studies (15, 16). Likewise, the unbalanced Th1/Th2 and Th17/Treg ratios have been reported in various autoimmune diseases (17, 18).

Th cells are the main producers of cytokines and play an important role in immune regulation and stimulation (16, 19). Therefore, serum cytokine levels often reflect the trend of T cell immunity. In this study, we demonstrated that women with positive aPL had significantly higher levels of Th1- and Th17-type pro-inflammatory cytokines, including IL-2 (Th1), TNF-α (Th1), IFN-γ (Th1), and IL-17A (Th17), and significantly lower level of Th2-type cytokines such as IL-4. Our data are consistent with a previous study, reporting significantly increased pro-inflammatory and prothrombotic cytokines in aPL-positive patients (20). These results indicate that aCL and/or aβ2GPI antibody-positive women undergoing IVF are in the pro-inflammatory state.

Recent studies report that TNF-α level in follicular fluid is negatively correlated with oocyte maturation and quality (21). Th1 cytokines, such as IFN‐γ and TNF‐α, directly promoted apoptosis and inhibited the proliferation of human granulosa cells (22). Additionally, aCL and/or aβ2GPI antibodies may directly affect the quality of oocytes. The aPL accumulate in the follicular fluid, bind to the surface of oocytes, and interfere with their development (23). Oocyte maturation and quality are important for the subsequent developmental potential of the embryos. Thus, Th1 and Th17 cell polarization and increased inflammatory cytokine production in aPL-positive women may have declined oocyte and embryo quality.

This study reports that aPL-positive women undergoing IVF have a lower implantation rate. In the process of embryo implantation, good-quality embryos and excellent uterine receptivity are crucial for clinical pregnancy. aPL can directly bind to the embryo, leading to retarded development, and also decrease uterine receptivity by inhibiting the decidualization of the endometrium (23–25). Moreover, IL-2 and IL-4 had opposite effects on embryo implantation. IL-2 may impair the implantation process, while IL-4 contributes to embryo implantation by inducing the expression of leukemia inhibitory factor (LIF) (26–28). Therefore, elevated IL-2 and decreased IL-4 observed in our study might further contribute to reduced embryo implantation.

It is generally accepted that immune homeostasis plays an important role in the embryo implantation and maintenance of pregnancy. Our results showed that aPL-positive women had Th1/Th2 and Th17/Treg immune imbalance, which is associate with adverse IVF outcomes, including lower clinical pregnancy rate, implantation rate, and take-home baby rate. In addition, it has been reported that Th1/Th2 and Th17/Treg imbalance can result in reproductive failures, such as spontaneous abortion and repeated implantation failure (RIF) (29, 30). Thus, unbalanced Th1/Th2 and Th17/Treg ratios and elevated serum inflammatory cytokines may be responsible for the impaired IVF outcomes in infertile women with aPL. Furthermore, the detrimental effect of aPL on trophoblast cells contributes to miscarriage. The aPL inhibit the cytotrophoblast to syncytiotrophoblast differentiation by adhesion to the phospholipid layer on the trophoblasts and promote the formation of microthrombi in placental vessels (31, 32).

This study demonstrated that women with multiple positive aPL had worse reproductive outcomes with IVF than those with single positive aPL, which was in agreement with previous studies that demonstrated that triple or double aPL positivity was the major risk factor for adverse pregnancy outcomes. In contrast, single aPL positivity carries a lower risk of obstetric complications (33). However, not all aPL confers the same degree of risk for adverse pregnancy outcomes, and controversies remain regarding which antibody is most relevant. Previously, lupus anticoagulant was reported to be more frequently associated with pregnancy complications than the other two aPL, including aCL and aβ2GPI. Contrarily, others reported that aCL or aβ2GPI were more closely associated with obstetrical complications than lupus anticoagulant (8, 34). High-quality studies with a large sample size are required.

Concerning the treatment of APS-complicated pregnancies, the current opinion of the first-line therapy tends to agree on LDA and LMWH therapy (35–37). Other therapies, such as hydroxychloroquine (HCQ), statins, drugs targeting B-cell, complement inhibitors, and intravenous immunoglobulins (IVIG), have also been reported to be beneficial to APS patients (38). However, there were no generalized recommendations for treating patients with positive aPL but not fulfilling the APS criteria, so-called non-criteria APS. A systematic review, including 5 studies involving 154 pregnancies, concluded that asymptomatic women with positive aPL did not benefit from LDA therapy concerning obstetric complications. However, in a EUROAPS cohort involving 650 non-criteria APS patients, both APS and non-criteria APS patients had similar fetal-maternal outcomes after treatment (39).

Moreover, treatment primarily aimed at effectively reducing aPL levels appeared to have little effect, although aPL was the key serological markers of APS. Recent studies have shown that the reduction or elimination of aPL by therapeutic regimen lasted longer but temporarily, and aPL quickly returned after stopping the treatment (3). In our study, applying LDA and hydroxychloroquine sulfate prior to the IVF cycles and the additional LMWH after the day of embryo transfer significantly improved IVF outcomes with increased perfect and available embryo rates, clinical pregnancy rate, implantation rate, and take-home baby rate. Improved reproductive outcomes may be attributed to improved embryo quality and controlled immune-inflammatory status. In addition, the local immune micromilieu is better adjusted to immune tolerance, resulting in improved endometrial receptivity and increased local blood supply. These changes promote embryo implantation while reducing the formation of microthrombi in the placenta, all of which play an important role in the implantation and maintenance of the embryo (40–42).

Whether the presence of aPL is associated with adverse IVF outcomes is still controversial and enigmatic. A recent meta-analysis reported that the presence of positive aPL neither decreased clinical pregnancy rate and live birth rate, nor increased miscarriage rate in women undergoing IVF (43). This discrepancy might be explained by the different predominant types of aPL; Sanmarco et al. reported that the prevalence of IgA class aCL and aβ2GPI was significantly higher than those of IgG or IgM class aPL (44). Hong et al., reported that the prevalence of IgG aβ2GPI was rather low and no woman had a positive IgG aCL antibody (45). However, in this study, the predominant types of aPL are IgG aCL and aβ2GPI. Recently, a prospective cohort study with a large sample size was performed to investigate whether the different types of aPL were associated with adverse IVF outcomes, demonstrating a detrimental impact of IgG and IgM aCL and IgG aβ2GPI on IVF outcomes (46). Thus, more studies focusing on antibody class, antigen specificity, antibody titers, and a presence of single vs. multiple antibody positivity are needed to further investigate the relationship between positive aPL and IVF outcomes.

Nevertheless, this study had some limitations. Firstly, the sample size is relatively small; hence, the data should be interpreted carefully, and clinical studies with a larger sample size are needed in the future. In addition, the heterogeneity of aPL-positive infertile patients is another limitation because some prothrombotic abnormalities may co-exist with aPL. aPL-positive women with and without prothrombotic abnormality need to be investigated in future studies.

## Conclusion

In conclusion, we report that aPL, such as aCL and/or aβ2GPI antibodies, are associated with adverse IVF outcomes, including lower clinical pregnancy, implantation, and take-home baby rates. Unbalanced immunological and inflammatory status, such as increased Th1/Th2 and Th17/Treg ratios and elevated serum inflammatory cytokines, may be responsible for the impaired IVF outcomes in infertile women with aPL. LDA and hydroxychloroquine sulfate combination treatment starting prior to the IVF cycle and LMWH after embryo transfer significantly improve the IVF outcomes. Although the incidence of aPL is low, early aPL assessment is recommended for infertile women undergoing IVF-ET cycles due to the significant impact of aPL on IVF outcomes. Once aPL is detected, including non-criteria aPL, optimal treatment should be considered before the IVF procedures.

## Data availability statement

The raw data supporting the conclusions of this article will be made available by the authors, without undue reservation.

## Ethics statement

The studies involving human participants were reviewed and approved by Ethics Committee of Anhui Provincial Hospital. The patients/participants provided their written informed consent to participate in this study.

## Author contributions

LW and XF worked on study design, data collection and analysis, and manuscript preparation. YW, FL, and YZ participated in data collection. LW and JK-K supervised the study, including study design, data analysis, and manuscript editing, and approved the version to be published.

## Funding

The study was supported by the National Natural Science Foundation of China: 82071650 and 82001641.

## Acknowledgments

The authors would like to thank the staff especially Shun Bai and Lan Guo in the Center for Reproductive Medicine and Laboratory for their assistance in data collection.

## Conflict of interest

The authors declare that the research was conducted in the absence of any commercial or financial relationships that could be construed as a potential conflict of interest.

## Publisher’s note

All claims expressed in this article are solely those of the authors and do not necessarily represent those of their affiliated organizations, or those of the publisher, the editors and the reviewers. Any product that may be evaluated in this article, or claim that may be made by its manufacturer, is not guaranteed or endorsed by the publisher.
